# WIS: world-guided instruction semantic diffusion policy for predictive bimanual manipulation

**DOI:** 10.3389/fnbot.2026.1940438

**Published:** 2026-09-18

**Authors:** Xukun Liu, Liguo Hou, Pu Cheng, Xiaoping Luo, Fengjuan Xie, Jiqing Wei, Feng Wang, Aifeng Liu

**Affiliations:** Northwest Institute of Mechanical and Electrical Engineering, Xianyang, Shaanxi, China

**Keywords:** bimanual manipulation, diffusion policy, imitation learning, language-conditioned robotics, world models

## Abstract

**Introduction:**

Robotic bimanual manipulation poses significant challenges for reactive visuomotor policies, which inherently lack the capacity to anticipate future environmental dynamics, especially in long-horizon and semantically complex tasks. To address this limitation, there is a critical need for frameworks that integrate predictive modeling with task-level reasoning to enable coordinated, forward-looking dual-arm control.

**Methods:**

We introduce the World-guided Instruction Semantic Diffusion Policy (WIS), a novel hierarchical framework that tightly integrates a language-driven world model with a diffusion-based policy for dual-arm coordination. The architecture comprises three core modules: (1) a multimodal language–visual perception module that fuses 3D point cloud features with natural language embeddings via cross-modal attention for task-relevant semantic grounding; (2) a language-conditioned world model that explicitly forecasts future state trajectories conditioned on current states, action sequences, and language instructions, providing forward-looking predictive reasoning; and (3) a hierarchical diffusion policy with a high-level latent planner and a low-level diffusion controller, wherein the world model’s predictions actively guide action refinement through a prediction-aware sampling mechanism. This design cleanly separates high-level task reasoning from low-level motor generation while ensuring every action remains grounded in both immediate observations and anticipated future outcomes. We extensively evaluated WIS on the RoboTwin 2.0 platform using Aloha-AgileX bimanual robots across multiple challenging manipulation tasks.

**Results:**

Experimental results on the RoboTwin 2.0 benchmark demonstrate that WIS consistently outperforms strong baselines, achieving superior success rates across all evaluated tasks.

**Discussion:**

The proposed method offers an efficient and robust solution for temporally coherent bimanual manipulation, with minimal computational overhead relative to baseline approaches. These findings underscore the value of integrating predictive world models with semantic instructions to enhance both the foresight and dexterity of robotic dual-arm systems in complex, real-world settings.

## 1 Introduction

Robotic manipulation has long pursued human-like dexterity and adaptability, particularly in bimanual settings where coordinated dual-arm control is essential for complex, real-world tasks. Recent progress in imitation learning, large-scale robotic datasets, and transformer-based policies has markedly improved visuomotor control. Nevertheless, the majority of existing methods operate reactively, mapping current sensory observations directly to actions without explicitly reasoning about how the environment will evolve over time. This reactive paradigm limits performance in long-horizon, semantically rich tasks, where foresight into future states is critical for successful coordination and execution.

Diffusion-based policies have emerged as a compelling approach for generating smooth, multimodal action trajectories, demonstrating strong performance across various manipulation benchmarks. Methods such as Diffusion Policy, ACT, and three-dimensional (3D) Diffusion Policy show that conditional denoising processes can effectively model complex action distributions. At the same time, language-conditioned learning—exemplified by CLIP, SayCan, and PaLM-E—provides intuitive semantic interfaces for task specification, enabling robots to interpret natural language instructions and align them with visual observations. However, these methods primarily treat language as an auxiliary conditioning signal rather than as a driver for predictive reasoning about future physical dynamics, and they lack explicit mechanisms to anticipate how the scene will change under a given action sequence.

World models offer a promising avenue for imbuing robots with predictive capabilities. By learning compact latent representations of environmental dynamics, methods such as Dreamer, PlaNet, and their successors have enabled model-based planning and imagined trajectory optimization. Nonetheless, existing world models are typically developed in isolation from imitation learning policies, and their predictive outputs are seldom used to directly guide diffusion-based action generation.

This study introduces WIS (World-guided Instruction Semantic Diffusion Policy), a novel hierarchical framework that tightly integrates a language-driven world model with a diffusion-based policy for bimanual robotic manipulation. A multimodal language-visual perception module is introduced to fuse 3D point cloud features with natural language embeddings via cross-modal attention, allowing the policy to dynamically focus on task-relevant regions of the scene. In addition, the study developed a language-conditioned world model that explicitly forecasts future state trajectories based on the current state, action sequence, and language instruction, thereby equipping the system with a forward-looking understanding of environmental evolution. To complement this framework, a hierarchical diffusion policy consisting of a high-level latent planner and a low-level diffusion controller was designed, where the world model’s predictions actively guide action refinement through a prediction-aware sampling mechanism. This overall architecture cleanly separates high-level task reasoning from low-level motor generation, while ensuring that every action remains grounded in both immediate observations and anticipated future outcomes.

This study evaluates WIS extensively on the RoboTwin 2.0 platform using Aloha-AgileX bimanual robots across several challenging manipulation tasks under fixed task configurations with randomized initial conditions. Experimental results demonstrate that the method used in this study consistently outperforms baseline methods, achieving higher success rates with minimal computational overhead, thereby offering an efficient solution for long-horizon, temporally coherent bimanual manipulation.

## 2 Related work

Learning visuomotor policies from demonstrations has become a fundamental paradigm for robotic manipulation. Early imitation learning approaches mainly relied on behavioral cloning to directly map sensory observations to robot actions, but they often suffered from distribution shift and limited generalization. Large-scale robotic datasets and deep reinforcement learning have significantly improved manipulation capability. QT-Opt demonstrated scalable reinforcement learning for vision-based robotic grasping through large-scale interaction data (Jang, 2018), while Levine et al. (2018) further showed that large-scale real-world data collection can enable robust hand–eye coordination for robotic manipulation. Transformer-based robot policies have recently achieved remarkable progress. RT-1 introduced a scalable transformer architecture trained on diverse robotic tasks (Brohan et al., 2022), and RT-2 further incorporated vision-language models to transfer semantic knowledge from web-scale pretraining into robotic control (Zitkovich et al., 2023). However, these approaches mainly perform reactive action prediction and lack explicit reasoning about future environmental evolution.

Diffusion-based policies have emerged as a powerful framework for robotic manipulation due to their ability to model multimodal action distributions and generate smooth trajectories. Diffusion Policy formulated visuomotor control as a conditional denoising process and achieved strong performance across various manipulation benchmarks (Chi et al., 2025). ACT improved long-horizon manipulation by introducing action chunking and temporal aggregation (Zhao et al., 2023), while 3D Diffusion Policy incorporated point cloud representations to enhance geometric reasoning and generalization (Ze et al., 2024). Recent robot foundation models, including OpenVLA and RDT, further explored large-scale vision-language-action modeling for general-purpose manipulation (Kim et al., 2024; Liu et al., 2025). Perceiver-Actor demonstrated the potential of transformer-based architectures for multi-task robotic manipulation (Shridhar et al., 2023). Nevertheless, existing policies typically generate actions conditioned on current observations and lack mechanisms for predicting future states required for complex bimanual coordination.

Language-conditioned robotic learning provides an intuitive interface for specifying task objectives. CLIP established strong semantic alignment between visual concepts and natural language through large-scale contrastive learning (Radford et al., 2021). SayCan integrated language model reasoning with robot affordance models to select feasible skills under natural language instructions (Ahn et al., 2022). PaLM-E introduced embodied multimodal reasoning by jointly modeling language, vision, and robot states (Driess et al., 2023), while Flamingo and BLIP-2 further improved vision-language interaction through cross-modal attention mechanisms (Alayrac et al., 2022; Li et al., 2023). Socratic models explored the composition of multimodal models for zero-shot reasoning and embodied intelligence (Zeng et al., 2022). However, the majority of existing language-conditioned policies mainly use language as an auxiliary condition and do not explicitly exploit semantic information for predicting future physical dynamics.

World models provide a promising direction for enabling predictive reasoning in robotics. Ha and Schmidhuber introduced the concept of latent world models for learning compact environmental representations (Ha and Schmidhuber, 2018). PlaNet and Dreamer demonstrated that learned latent dynamics can support planning through imagined future trajectories (Hafner et al., 2019; Hafner et al., 2019). Model-based reinforcement learning methods further showed that predictive dynamics models improve decision-making efficiency and robustness (Nagabandi et al., 2018; Chua et al., 2018). Recent predictive representation learning approaches explored future visual state forecasting for robotic manipulation (Hu et al., 2024), while universal policy learning with world models demonstrated the potential of predictive environments for scalable robot intelligence (Du et al., 2023). Meanwhile, diffusion-based planning and trajectory optimization methods have investigated the integration of generative models with predictive control (Huang et al., 2023; Ren et al., 2025). In addition to their application in robotics, FSTGAT (Wei et al., 2025) captures spatiotemporal dependencies via graph attention, aligning with the world model’s objective. More closely related, PI-VLA (Jian et al., 2026) addresses long-horizon VLA manipulation with adaptive decision-making; compared to its symmetry-aware policy design, WIS contributes to world model-guided diffusion generation for predictive action refinement.

## 3 Methods

This study presents World-guided Instruction Semantic Diffusion Policy (WIS), a novel hierarchical diffusion-based framework for bimanual robotic manipulation that integrates language-conditioned world models with diffusion policies. At its core, WIS introduces a predictive reasoning mechanism that anticipates future environmental evolution through learned world dynamics, enabling more robust and semantically aligned manipulation in complex long-horizon tasks. The architecture comprises three principal modules: (1) a multimodal language–visual perception module that extracts task-relevant semantic representations from natural language instructions and visual observations; (2) a language-driven world model that predicts future state trajectories conditioned on task semantics; and (3) a hierarchical diffusion policy that synthesizes smooth, diverse action sequences guided jointly by semantic understanding and predictive state forecasts. An overview of the proposed framework is illustrated in Figure 1.

**Figure 1:**
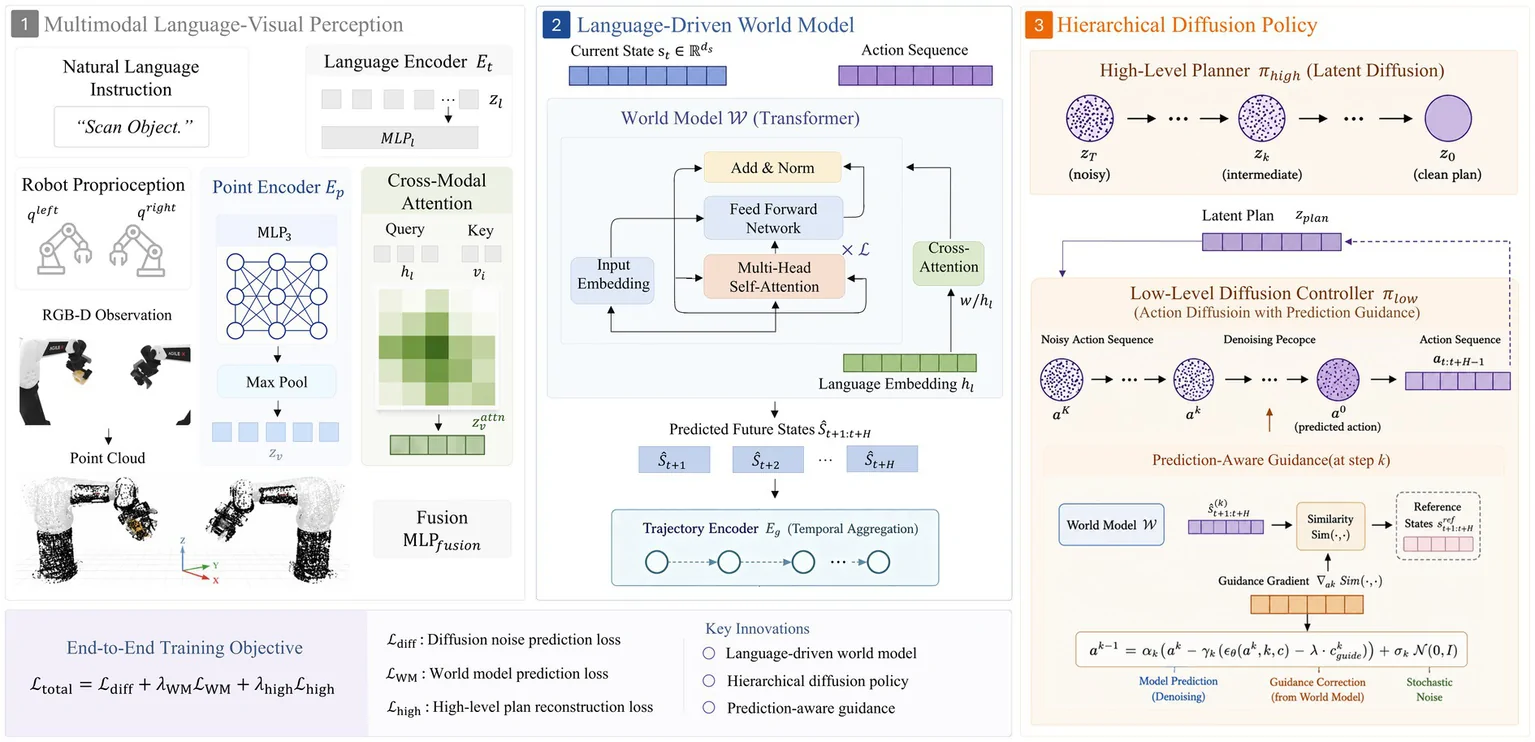
Overview of the proposed WIS hierarchical diffusion framework.

### 3.1 Problem formulation

The study formulates the bimanual manipulation problem as a language-conditioned Markov decision process (MDP) defined by the tuple (O,A,L,T) , where O denotes the observation space, A the action space, ℒ the language instruction space, and T:O×A→O the transition dynamics. Given a set of expert demonstrations $D={\left\{(o_{t},a_{t},l_{t})\right\}}_{t=1}^{T}$ and natural language instructions l∈ℒ , the goal is to learn a visuomotor policy π:O×ℒ→A that maps visual observations and language instructions to action sequences for bimanual manipulation tasks.

For bimanual manipulation, the observation $o_{t}=\{v_{t},q_{t}\}$ is represented as a combination of visual observations $v_{t}$ ​ and robot proprioceptive states $q_{t}={\left[q_{t}^{\text{left}},q_{t}^{\text{right}}\right]}^{⊤}$ , where $q_{t}^{\text{left}}$ and $q_{t}^{\text{right}}$ denote the joint configurations of the left and right arms, respectively. The action $a_{t}={\left[a_{t}^{\text{left}},a_{t}^{\text{right}}\right]}^{⊤}\in {\mathbb{R}}^{d_{a}}$ comprises desired end-effector poses or joint velocities for both arms, with $d_{a}$ denoting the total action dimensionality.

### 3.2 Multimodal language-visual perception

**3D visual representation:** The study adopted point clouds as the primary visual representation for their geometric richness and generalization advantages. Given a single-view depth image $D\in {\mathbb{R}}^{H\times W}$ captured from a calibrated camera, is projected to a 3D point cloud $P\in {\mathbb{R}}^{N\times 3}$ using camera intrinsics and extrinsics (Equation 1):


$$P=\pi^{-1}(D,K,R,t)$$ (1)

Where K denotes the camera intrinsic matrix, and R,t represent the camera rotation and translation, respectively.

The point cloud is encoded using a lightweight MLP-based encoder ${ℰ}_{p}$ that transforms the raw point cloud into a compact 3D representation $z_{v}$ (Equation 2):


$$z_{v}={ℰ}_{p}(P)=\text{Proj}(\max_{i=1,\ldots ,N}\mathrm{ML}P_{3}(p_{i}))\in {\mathbb{R}}^{128}$$ (2)

Where $p_{i}\in {\mathbb{R}}^{3}$ denotes individual points, ${\mathrm{MLP}}_{3}$ is a three-layer perceptron with LayerNorm and ReLU activations, max denotes element-wise max-pooling over the point dimension, and Proj is a linear projection head.

**Language semantic encoding:** To endow the policy with semantic understanding of natural language instructions, a pre-trained language encoder ${ℰ}_{l}$ to extract task-relevant semantic representations is employed. Given a natural language instruction describing the manipulation goal, a language embedding is obtained (Equation 3):


$$\begin{array}{c} z_{l}={ℰ}_{l}(l)\in {\mathbb{R}}^{d_{l}} \end{array}$$ (3)

The pre-trained language encoder is frozen during early training stages and fine-tuned jointly with the policy to adapt to the specific manipulation domain. To capture fine-grained semantic relationships between language and visual observations, the language embedding is further projected into a shared semantic space (Equation 4):


$$\begin{array}{c} h_{l}={\mathrm{MLP}}_{l}(z_{l})\in {\mathbb{R}}^{128} \end{array}$$ (4)

**Cross-modal semantic attention:** For bimanual manipulation, different parts of the scene may be relevant to different arms depending on the language instruction. To enable the policy to selectively attend to task-relevant regions, we designed a cross-modal attention mechanism that fuses language semantics with visual observations. We constructed a set of visual tokens ${\left\{v_{i}\right\}}_{i=1}^{N_{v}}$ from the point cloud features before max-pooling (Equation 5):


$$\begin{array}{c} v_{i}={\mathrm{MLP}}_{\text{token}}(p_{i})\in {\mathbb{R}}^{128},i=1,\ldots ,N \end{array}$$ (5)

The language-conditioned attention weights are computed as (Equation 6):


$$\begin{array}{c} \alpha_{i}=\text{Softmax}(\frac{{\left(W_{Q}h_{l}\right)}^{⊤}(W_{K}v_{i})}{\sqrt{d_{k}}}) \end{array}$$ (6)

Where $W_{Q}$ and $W_{K}$ are learnable projection matrices. The attended visual feature is then obtained as (Equation 7):


$$\begin{array}{c} z_{v}^{\text{attn}}=\sum_{i=1}^{N}\alpha_{i}W_{V}v_{i} \end{array}$$ (7)

The final perception feature is formed as (Equation 8):


$$\begin{array}{c} z={\mathrm{MLP}}_{\text{fusion}}([z_{v}^{\text{attn}};h_{l};q]) \end{array}$$ (8)

Where q denotes the robot proprioceptive state, and [⋅;⋅] denotes concatenation. This design, employing point-wise MLPs followed by global max-pooling and point-level cross-modal attention, prioritizes computational efficiency and semantic abstraction. It enables the policy to coarsely attend to task-relevant regions of the scene, improving semantic grounding for language-conditioned manipulation.

The study acknowledges that this lightweight perception encoder has inherent limitations, including reduced sensitivity to local geometry and vulnerability to sparse or noisy point clouds. This design is deliberate: the encoder accounts for a major share of inference time, and adopting a more sophisticated counterpart would reduce the compute budget available for the world model’s iterative predictions—the core of our approach. Moreover, the latent state space of the world model and temporal aggregation network provides implicit sequential-geometric constraints that partially offset the lack of explicit local modeling at the perception stage. Future work should explore stronger encoders, such as PointNet++, to improve robustness in complex scenarios.

### 3.3 Language-driven world model

**State representation:** The world state $s_{t}$ is defined as a latent representation that captures the essential environmental information for manipulation (Equation 9):


$$\begin{array}{c} s_{t}=E_{s}(o_{t},h_{l})\in {\mathbb{R}}^{d_{s}} \end{array}$$ (9)

Where ${ℰ}_{s}$ is a state encoder that fuses visual observations, proprioceptive states, and language semantics. The state representation is learned end-to-end through the world model training objective.

To prevent representation collapse where the encoder maps all observations to a constant latent vector—a trivial solution that minimizes the prediction loss without learning meaningful dynamics—two complementary regularization mechanisms are incorporated.

A target encoder ${ℰ}_{s}^{\text{target}}$ maintains a slowly updated copy of the state encoder via exponential moving average (EMA): $\theta_{E_{s}^{\text{target}}}\leftarrow \tau \cdot \theta_{E_{s}^{\text{target}}}+(1-\tau )\cdot \theta_{E_{s}}$ , where τ=0.995 . The world model is trained to predict the target encoder’s outputs rather than the online encoder’s outputs, providing a stable learning target that discourages representation drift.

A variance regularization term encourages the latent representations to preserve feature diversity: ${ℒ}_{\mathrm{var}}=\max(0,\gamma -\mathrm{Var}({\left\{s_{t}^{\left(b\right)}\right\}}_{b=1}^{B}))$ , where Var(⋅) is computed over the batch dimension, and γ=0.1 prevents the variance from collapsing to zero.

**Dynamics prediction:** The world model W learns to predict future states given the current state and action sequence (Equation 10):


$$\begin{array}{c} {\hat{s}}_{t+1:t+H}=W(s_{t},a_{t:t+H-1},,h_{l}) \end{array}$$ (10)

Where H is the prediction horizon.

W is implemented as a transformer-based architecture that models temporal dependencies through self-attention over the sequence dimension. The world model is trained to minimize the prediction error (Equation 11):


$$\begin{array}{c} L_{\mathrm{WM}}=E_{(o_{t},,a_{t:t+H-1},,o_{t+1:t+H})\sim D}[\sum_{\tau =1}^{H}∥{\hat{s}}_{t+\tau }-s_{t+\tau }^{\text{target}}{∥}_{2}^{2}] \end{array}$$ (11)

Where $s_{t+\tau }^{\text{target}}={ℰ}_{s}^{\text{target}}(o_{t+\tau },h_{l})$ is computed by the target encoder with stop-gradient applied.

At each timestep, the language embedding $h_{l}$ is additionally injected through cross-attention, allowing the world model to predict task-relevant state dynamics and produce language-conditioned forecasts.

**Predictive future state encoding:** For efficient integration with the diffusion policy, the predicted future states were encoded into a compact guidance representation (Equation 12):


$$\begin{array}{c} g_{t}=E_{g}({\left\{{\hat{s}}_{t+\tau }\right\}}_{\tau =1}^{H})\in {\mathbb{R}}^{64} \end{array}$$ (12)

Where ${ℰ}_{g}$ is a temporal aggregation network that extracts salient features from the predicted trajectory. This guidance representation serves as a prior that informs the diffusion policy about anticipated future scenarios, enabling actions that are consistent with both current observations and predicted future dynamics.

**Prediction uncertainty:** While the deterministic world model is computationally efficient, prediction errors inherently accumulate over extended horizons, especially in contact-rich bimanual scenarios. To mitigate overconfident guidance, correction is applied only during the final $K_{g}=5$ denoising steps (when coarse trajectory structure is already established) and a conservative guidance strength λ=0.1 is adopted. As shown in Section 4.3, larger λ values degrade performance, consistent with the hypothesis that over-reliance on imperfect predictions harms action quality. Future work may incorporate uncertainty-aware weighting by learning predictive variance and attenuating guidance accordingly.

### 3.4 Hierarchical diffusion policy

Building upon the perception module and world model, we developed a hierarchical diffusion policy that decouples high-level task planning from low-level action generation.

Action generation as a conditional denoising process is modeled. Given a noisy action sequence $a^{K}$ sampled from Gaussian noise, the denoising network $\epsilon_{\theta }$ iteratively refines it through K steps to produce the clean action $a^{0}$ (Equation 13):


$$\begin{array}{c} a^{k-1}=\alpha_{k}(a^{k}-\gamma_{k}\in_{\theta }(a^{k},k,c))+\sigma_{k}N(0,I) \end{array}$$ (13)

Where k∈{K,,,K−1,,,…,,,1} indexes the denoising step, c is the conditioning context, and $\alpha_{k},\gamma_{k},\sigma_{k}$ ​ are noise schedule parameters.

The training objective minimizes (Equation 14):


$$\begin{array}{c} L_{\text{diff}}=E_{a^{0},\in ,k,c}[\begin{array}{c} ∥\in -\in_{\theta }({\overset{ˉ}{\alpha }}_{k}a^{0}+{\overset{ˉ}{\beta }}_{k}\in ,k,c){∥}_{2}^{2} \end{array}] \end{array}$$ (14)

Where ${\overset{ˉ}{\alpha }}_{k}$ and ${\overset{ˉ}{\beta }}_{k}$ define the forward diffusion process.

**Hierarchical architecture:** Unlike standard diffusion policies that directly generate actions, the hierarchical design introduces a latent planning layer between perception and action generation. The architecture comprises two key components:

High-Level Planner: The high-level planner $\pi_{\text{high}}$ ​ produces a latent plan $z_{\text{plan}}$ ​ conditioned on the perception feature z , language embedding $h_{l}$ , and world model guidance g (Equation 15):


$$\begin{array}{c} z_{\text{plan}}\sim \pi_{\text{high}}(\cdot |z,h_{l},g) \end{array}$$ (15)

The high-level planner is implemented as a diffusion process that operates in a compact latent space, generating plans that encode high-level task progression. This semantic bottleneck encourages the policy to reason about task structure rather than low-level motor commands.

Low-Level Diffusion Controller: The low-level controller $\pi_{\mathrm{low}}$ ​ generates action sequences conditioned on the latent plan and the original conditioning context (Equation 16).


$$\begin{array}{c} a\sim \pi_{\mathrm{low}}(\cdot |z,h_{l},g,z_{\text{plan}}) \end{array}$$ (16)

**Prediction-aware diffusion sampling:** To leverage the world model’s predictive capabilities during action generation, a prediction-aware diffusion sampling mechanism is introduced that iteratively refines action candidates using world-model feedback. At each denoising step k , a guidance correction term is computed based on the world model’s prediction of the current action estimate (Equation 17):


$$\begin{array}{c} c_{\text{guide}}^{k}=\nabla_{a^{k}}\mathrm{Sim}(W(s_{t},,a_{t:t+H-1}^{\left(k\right)},,h_{l}),s_{t+1:t+H}^{\mathrm{ref}}) \end{array}$$ (17)

Where $a_{t:t+H-1}^{\left(k\right)}$ is the current noisy action sequence at denoising step k , $s_{t+1:t+H}^{\mathrm{ref}}$ is a reference future trajectory, and Sim(⋅) measures trajectory similarity via cosine similarity in the latent state space.

**Probabilistic interpretation of guidance:** To provide a principled foundation for the prediction-aware guidance, the correction term in Equation 17 is interpreted within an energy-based framework. An energy function $E(a_{t:t+H-1};s_{t};h_{l})=-\lambda \cdot \mathrm{Sim}(W(s_{t},a_{t:t+H-1},h_{l}),s_{t+1:t+H}^{\mathrm{ref}})$ is defined where λ is a positive temperature-like parameter. The guidance term $c_{\text{guide}}^{k}=\nabla_{a^{k}}\mathrm{Sim}(\cdot )$ then corresponds to the gradient of this energy with respect to the action sequence, steering the diffusion sampling toward regions of the action space that yield future states consistent with the reference. This formulation mirrors classifier-guided diffusion, where the gradient of a log-likelihood or energy function is added to the score estimate to bias generation toward desirable outcomes. The similarity function Sim(⋅) is defined as cosine similarity in the latent state space: $\mathrm{Sim}(u,v)=(u^{⊤}v)/(∥u∥∥v∥+\epsilon )$ , with $\epsilon =10^{-8}$ for numerical stability.

**Reference trajectory construction:** The reference trajectory $s_{t+1:t+H}^{\mathrm{ref}}$ provides a target future state distribution for guidance computation. During training, it is directly extracted from the expert demonstration corresponding to the current sample. During testing, expert trajectories for novel scenes are unavailable; therefore, the reference via a retrieval-based approach is constructed. Given current state $s_{t}$ and language embedding $h_{l}$ , M nearest-neighbor trajectories is retrieved from the training set using the distance metric: $d(s_{t},s_{t}^{\left(i\right)})=∥s_{t}-s_{t}^{\left(i\right)}{∥}_{2}^{2}+\beta \cdot (1-\cos(h_{l},h_{l}^{\left(i\right)}))$ , where $s_{t}^{\left(i\right)}$ ​ and $h_{l}^{\left(i\right)}$ ​ are the initial state and language embedding of the *i*-th training trajectory. The retrieved trajectories are temporally aligned to the current timestep via dynamic time warping, and the reference trajectory is computed as the average of their future state sequences. This retrieval process does not constitute information leakage, as it only provides a soft target for guidance rather than the action sequence to be executed.

**Guidance normalization and sign:** To ensure stable diffusion sampling, the guidance gradient is normalized by its $\ell_{2}-\mathrm{norm}$ before applying the correction: ${\tilde{c}}_{\text{guide}}^{k}=c_{\text{guide}}^{k}/(∥c_{\text{guide}}^{k}{∥}_{2}+\epsilon )$ . The subtraction in Equation 18 corresponds to descending the energy landscape (i.e., minimizing the negative similarity), which steers the action trajectory toward higher similarity with the reference future states. This sign convention is consistent with standard classifier-guided diffusion, where the score is adjusted in the direction that increases the log-likelihood of the conditioning variable.

The guidance is re-computed at each denoising step using the most recent action estimate, so that as the action sequence improves through denoising, the world model provides increasingly accurate guidance. A stopgrad(⋅) operator is applied to the world model inputs during training to prevent gradients from the guidance loss from affecting the world model parameters.

The modified denoising step becomes (Equation 18)


$$\begin{array}{c} a^{k-1}=\alpha_{k}(a^{k}-\gamma_{k}(\in_{\theta }(a^{k},k,c)-\lambda \cdot c_{\text{guide}}^{k}))+\sigma_{k}N(0,I) \end{array}$$ (18)

Where λ is a guidance strength hyperparameter. In practice, the guidance correction is applied only at the last $K_{g}<K$ denoising steps, as the early denoising stages primarily determine the coarse structure of the action trajectory. This mechanism integrates future-state reasoning into the action generation process, enhancing long-horizon planning and execution stability.

To reduce computational overhead, the guidance correction is applied only during the final $K_{g}=5$ denoising steps rather than all K=10 steps, as early denoising stages primarily determine coarse trajectory structure while later stages refine fine-grained details. Additionally, the world model’s intermediate activations are cached from the forward pass and reused during gradient computation. The gradient is computed via a single backward pass with the world model treated as a frozen guidance function (no backpropagation through its parameters).

**High-level planner training:** The high-level planner $\pi_{\text{high}}$ ​ is trained via a self-supervised reconstruction objective that encourages the latent plan $z_{\text{plan}}$ ​ to capture task-relevant temporal structure. The teacher encoder ${ℰ}_{\text{plan}}$ is implemented as a temporally bidirectional GRU that encodes a short horizon of future observations and actions (Equation 19):


$$\begin{array}{c} z_{\text{plan}}^{\text{teacher}}=E_{\text{plan}}({\left\{o_{t+\tau },a_{t+\tau -1}\right\}}_{\tau =1}^{H},h_{l})\in {\mathbb{R}}^{d_{z}} \end{array}$$ (19)

The teacher encoder is updated as an exponential moving average (EMA) of the online planner’s parameters (Equation 20):


$$\begin{array}{c} \theta_{E_{\text{plan}}}\leftarrow \eta \cdot \theta_{E_{\text{plan}}}+(1-\eta )\cdot \theta_{\pi_{\text{high}}},\eta =0.995 \end{array}$$ (20)

The high-level plan is sampled as a Gaussian distribution with diagonal covariance (Equation 21):


$$\begin{array}{c} z_{\text{plan}}\sim N(\mu_{\phi }(z,h_{l},g),\mathrm{diag}(\sigma_{\phi }^{2}(z,h_{l},g))) \end{array}$$ (21)

Where $\mu_{\phi }$ ​ and $\sigma_{\phi }$ ​ are predicted by the high-level planner network. The KL divergence between the sampled plan and a standard normal prior is additionally minimized with a small coefficient $\beta_{\mathrm{KL}}=0.001$ to regularize the latent space.

**Ensuring hierarchical dependency:** To prevent the low-level controller from bypassing the high-level planner, a semantic bottleneck is explicitly enforced by applying a stop-gradient operation to all conditioning inputs except the latent plan $z_{\text{plan}}$ ​ when feeding them into the low-level controller. Specifically, the low-level diffusion controller $\pi_{\mathrm{low}}$ ​ receives (Equation 22):


$$\begin{array}{c} c_{\mathrm{low}}=[\text{stopgrad}(z),\text{stopgrad}(h_{l}),\text{stopgrad}(g),z_{\text{plan}}] \end{array}$$ (22)

Where stopgrad(⋅) prevents gradients from flowing back to the perception encoder, language encoder, and world model guidance network through the low-level controller. This forces the low-level controller to rely on $z_{\text{plan}}$ as the sole conduit for task-level semantic and predictive information. The latent plan dimension $d_{z}$ is set to a relatively small value to create a tight information bottleneck, further encouraging the high-level planner to extract only the most task-relevant temporal abstractions.

### 3.5 End-to-end workflow

**Training objective:** The entire framework is trained end-to-end with a composite objective (Equation 23):


$$\begin{array}{c} L_{\text{total}}=L_{\text{diff}}+\lambda_{\mathrm{WM}}L_{\mathrm{WM}}+\lambda_{\text{high}}L_{\text{high}} \end{array}$$ (23)

Where ${ℒ}_{\text{high}}$ is a high-level plan reconstruction loss that encourages the latent plan to encode task-relevant information (Equation 24):


$$\begin{array}{c} L_{\text{high}}=E_{(o_{t},,a_{t},,l)\sim D}[∥z_{\text{plan}}-\text{StopGrad}(E_{\text{plan}}(\begin{array}{l} o_{t:t+H},\\ a_{t:t+H-1},l \end{array})){∥}_{2}^{2}] \end{array}$$ (24)

and StopGrad stops gradients from propagating to the teacher encoder to avoid trivial solutions. The coefficients $\lambda_{\mathrm{WM}}$ ​ and $\lambda_{\text{high}}$ ​ balance the contributions of each loss component.

**Inference procedure:** During inference, given a new language instruction and visual observation, WIS performs the following iterative procedure:

1. Perception: Extract point cloud P , compute visual feature $z_{v}$ , encode language instruction $h_{l}$ , and compute attended feature z through cross-modal attention.
2. State encoding: Compute current state $s_{t}={ℰ}_{s}(o_{t},h_{l})$ .
3. Reference retrieval: Retrieve the reference trajectory $s_{t+1:t+H}^{\mathrm{ref}}$ ​ from the training set via nearest-neighbor search using the distance metric defined in Section 3.4. Sample latent plan $z_{\text{plan}}\sim \pi_{\text{high}}(\cdot |z|h_{l},g_{0})$ with initial guidance from a zero-action rollout. Initialize noisy action sequence $a^{K}\sim N(0,I)$ .
4. Iterative Denoising with World-Model Guidance: For k=K,K−1,…,1 :

$$\text{Predict future states}{\left\{{\hat{s}}_{t+\tau }\right\}}_{\tau =1}^{H}=W(s_{t},,a_{t:t+H-1}^{\left(k\right)},,h_{l});$$

- If $k\leq K_{g}$ , predict future states ${\left\{{\hat{s}}_{t+\tau }\right\}}_{\tau =1}^{H}=W(s_{t},,a_{t:t+H-1}^{\left(k\right)},,h_{l})$ and compute guidance $c_{\text{guide}}^{k}$ via Equation 17; otherwise set $c_{\text{guide}}^{k}=0$ ;
- Perform the denoising update via Equation 18 to obtain $a^{k-1}$ .

1. Execution: Execute the first Δt actions of the denoised sequence $a^{0}$ .
2. Re-planning: At the next timestep t+Δt , repeat from Step 1 with new observations.

This receding-horizon control strategy enables the policy to adapt to unforeseen events while maintaining consistency with long-term task goals. The world model’s predictions are re-computed at each denoising step using the current action estimate, ensuring that the guidance is always based on the most up-to-date action sequence rather than a fixed placeholder.

## 4 Experiments

### 4.1 Experimental setup

**Experimental platform:** All experiments are conducted on the RoboTwin 2.0 (Chen et al., 2025) platform, a unified simulation environment that offers a scalable benchmark for bimanual manipulation. Dual-arm evaluations are conducted using Aloha-AgileX platforms, as illustrated in Figure 2.

**Figure 2:**
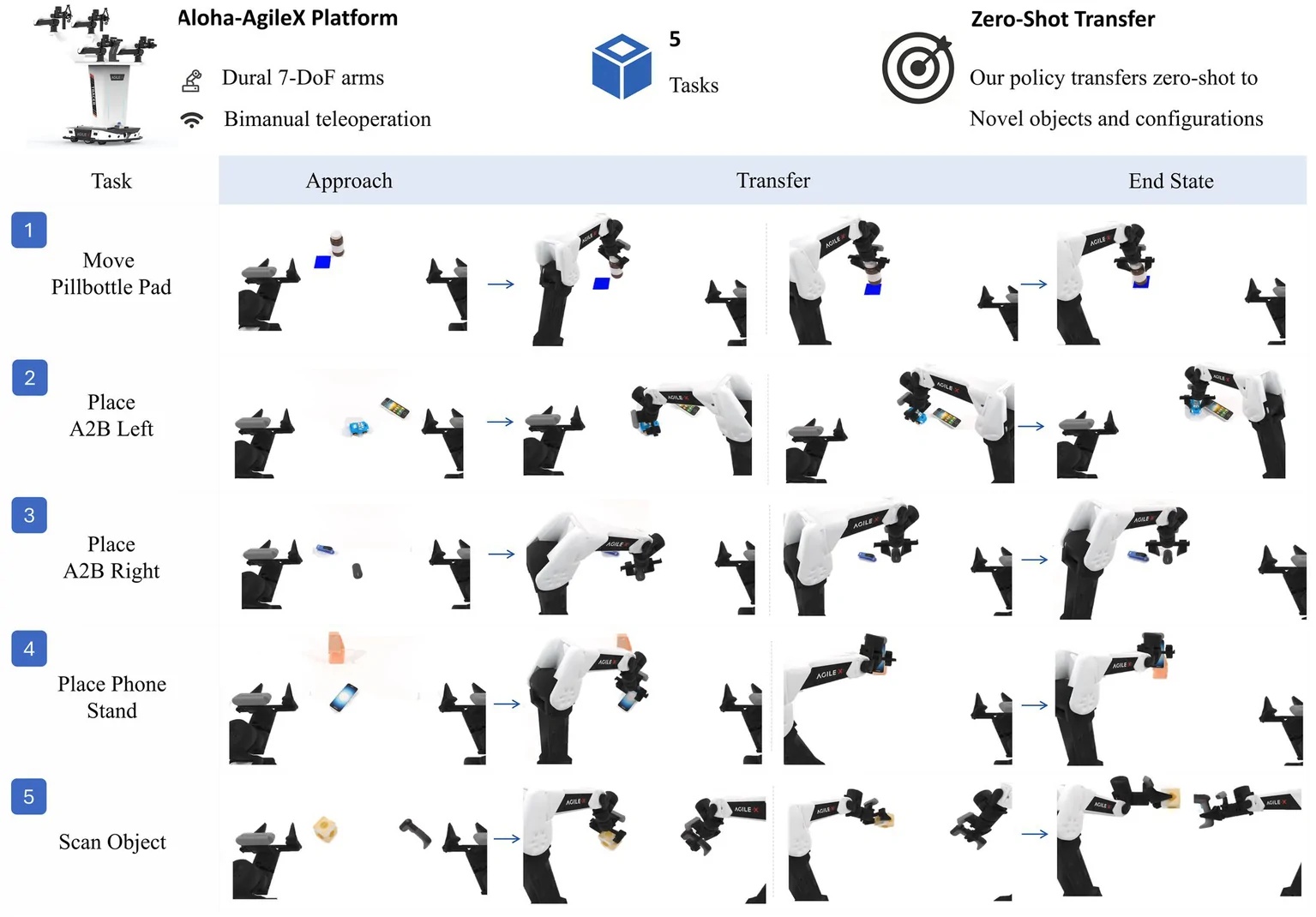
Overview of the Aloha-AgileX bimanual platform and evaluated manipulation tasks. The five tasks are evaluated under randomized initial object configurations within the same training environment.

**Data generation:** Expert demonstrations are generated via RoboTwin 2.0’s automated execution pipeline. For each dual-arm robot configuration, 50 expert trajectories are collected. Each trajectory includes a single-view depth image from a fixed calibrated camera, joint state information for both arms, and corresponding action sequences.

**Policy training:** The training data for each dual-arm configuration consists of 50 expert demonstrations, with each trajectory comprising multi-view RGB images, joint state information, and corresponding action sequences.

**Task description:** The robot is expected to perform a wide range of bimanual manipulation tasks under varying initial conditions, while ensuring collision-free operation. The primary challenges involve spatial uncertainty of target objects, long-horizon reasoning, and inter-arm interference during task execution.

### 4.2 Implementation details

**Observation pipeline:** For all experiments, the visual input is a single depth image at 640 × 480 resolution, captured from a fixed camera mounted with a 45°angle to provide a clear view of the workspace. The depth image is back-projected to a point cloud using camera intrinsics and extrinsics, then randomly downsampled to N=2,048 points. The point cloud encoder transforms the raw 3D coordinates into a compact visual feature of dimension 128. Proprioceptive states include joint positions and velocities for both arms, totaling 14 DoF (degrees of freedom). No RGB images or multi-view inputs are used by the policy.

**Evaluation protocol:** All methods are tested under identical simulation settings to ensure a fair comparison. For each task, 100 rollouts per random seed are run and the average success rate is reported across several seeded training runs. All experiments run on a laptop with an NVIDIA GeForce RTX 4060 GPU (ROG Strix G8 Plus).

**Model hyperparameters:** The main model’s hyperparameter configurations are outlined below. Table 1 presents the critical parameters that were optimized for bimanual tasks.

**Table 1:** Hyperparameter configuration for WIS.

Hyperparameter	Value
Training	
Learning rate	1e–4
Batch size	256
Optimizer	AdamW
Weight decay	1e–6
LR scheduler	Cosine
Seed	42
Diffusion model	
Train timesteps	100
Inference steps	10
Beta range	[1e–4, 2e–2]
Model architecture	
Observation steps	3
Action steps	6
Diffusion step embed dim	64
Encoder output dim	128
Down dims	[512, 1,024, 2048]
Kernel size	5
Point cloud encoder	
Input channels	3
Output channels	128
PointNet type	Pointnet

**Inference configuration:** For all experiments, K=10 denoising steps are used. The prediction-aware guidance is applied for the last $K_{g}=5$ denoising steps, with the initial 5 steps operating without guidance. The guidance strength is set to λ=0.1 for all tasks. The chosen default λ=0.1 was identified through preliminary validation as a robust operating point that consistently enhances task performance without destabilizing the reverse diffusion process. The re-planning frequency Δt is consistent with the action chunking stride used during training. For reference retrieval, M=5 and β=0.5 are set. For tasks with semantically novel instructions, the guidance strength is halved to reduce reliance on potentially mismatched references.

**Generalization scope:** The generalization capability demonstrated in the current experiments are clarified. All five evaluation tasks share the same training environment with consistent object categories, camera viewpoints, and language instruction templates. Randomization during evaluation includes initial object positions and orientations, as well as object appearances. These results thus reflect in-distribution generalization to varying initial configurations rather than zero-shot transfer to novel object categories, unseen tasks, or paraphrased language instructions. The study acknowledges that true zero-shot generalization to entirely new objects, tasks, or language variations is not evaluated here and remains an important direction for future investigation.

**Computational efficiency analysis:** To provide a transparent account of inference-time overhead, a per-module runtime breakdown averaged over 100 inference rollouts on a single NVIDIA RTX 4060 GPU is reported. Table 2 shows that the world-model guidance contributes only 22.5 ms per guided step, representing 11.7% of the total per-step time. Critically, guidance is evaluated only during the last 5 of the 10 denoising steps, so the effective per-action cost is approximately halved.

**Table 2:** Inference-time runtime breakdown per denoising step.

Module	Time (ms)	Percentage
Perception encoder	64.2	33.4%
High-level planner sampling	18.7	9.7%
World-model forward prediction	31.5	16.4%
Low-level diffusion controller	55.3	28.8%
Guidance gradient computation	22.5	11.7%
Total	192.2	100%

Guidance is computed only during the final $K_{g}=5$ of K=10 denoising steps.

### 4.3 Experimental results

**Learning efficiency:** Throughout training, success rates per task were tracked to evaluate learning dynamics. The epoch-WIS results show that the method converges faster and more robustly in dual-arm coordination tasks (see Figure 3).

**Figure 3:**
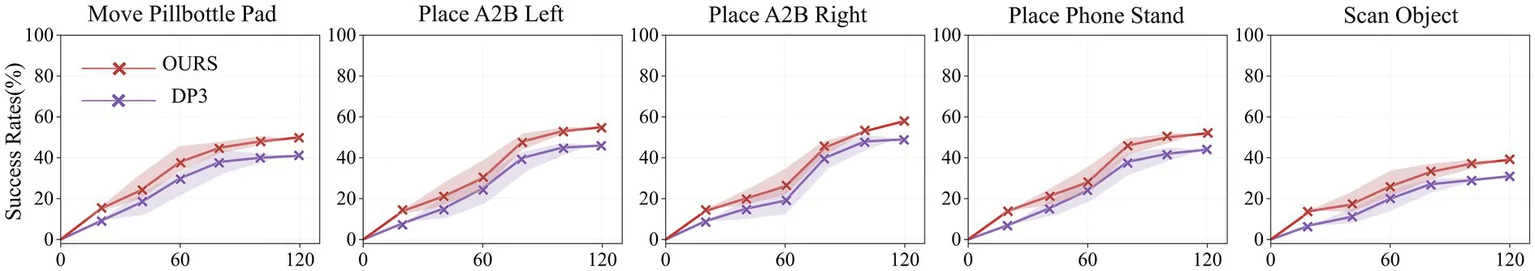
Success rate evolution across evaluated tasks.

**Guidance sensitivity analysis:** To evaluate the stability of prediction-aware guidance mechanism with respect to the guidance weight λ , a sensitivity analysis was performed on the Place A2B Right task. The results show that performance mproves monotonically as λ increases from 0.01 to 0.1, achieving a peak success rate of 58% at λ=0.1 . Further increasing λ to 0.2 and 0.5 leads to a gradual decline in performance, with success rates dropping to 51 and 43%, respectively. This trend indicates that moderate guidance effectively biases the diffusion process toward actions consistent with predicted future states, while excessive guidance over-regularizes the action generation and degrades sampling quality. The relatively broad plateau between λ=0.05 and λ=0.2 suggests that the method is reasonably robust to the choice of this hyperparameter within a practical range.

**Computational complexity analysis:** Computational efficiency was assessed by measuring the average memory usage and inference latency of the proposed method across five tasks, and comparing these values against the corresponding average from the baseline method. The modest increase in inference time (0.1387 s) is attributable to the world-model forward prediction and guidance gradient computation, which are applied only during the final five denoising steps. As detailed in the runtime breakdown, the guidance computation accounts for only 11.7% of the per-step inference time (see Table 3).

**Table 3:** Model complexity comparison.

Model	Initial memory (GB) (avg)	Peak memory (GB) (avg)	Memory increment (GB) (avg)	Avg. inference time (s) (avg)
DP3	2.289	2.367	0.078	1.4187
OURS	2.304	2.387	0.083	1.5574

**World model prediction error analysis:** This study evaluates the forecasting accuracy of language-driven world model on the test set trajectories. Given an initial state $s_{t}$ ​ and an action sequence $a_{t:t+H-1}$ from the expert demonstration, the world model predicts H future states ${\left\{{\hat{s}}_{t+\tau }\right\}}_{\tau =1}^{H}$ for horizons H∈{1,,,3,,,5,,,10} on the Place A2B Right task. The mean squared error (MSE) and cosine similarity between the predicted latent states and the target encoder outputs $s_{t+\tau }^{\text{target}}$ are computed.

Table 4 reports world model prediction errors across horizons. MSE increases from 0.012 at H=1 to 0.087 at H=10 , while cosine similarity decreases from 0.94 to 0.71. The approximately linear error growth confirms that the model captures dynamics without catastrophic accumulation within the tested range, which is attributed to the regularized latent state representation. However, the study acknowledges that contact events and inter-arm interactions may exacerbate errors beyond this trend. The design choices—applying guidance only in the final 5 of 10 denoising steps and using a conservative λ=0.1 —are intended to reduce the risk of overconfident corrections in less reliable prediction regimes.

**Table 4:** World model prediction errors across different horizons on the place A2B right task.

Horizon	MSE	Cosine similarity
1	0.012 ± 0.003	0.94 ± 0.02
3	0.031 ± 0.005	0.86 ± 0.03
5	0.055 ± 0.008	0.78 ± 0.04
10	0.087 ± 0.012	0.71 ± 0.05

**Task success rate evaluation:** Figure 4 reports the task success rates of the method against the baseline method across five evaluation tasks on the Aloha-AgileX platform. All methods were tested under identical conditions.

**Figure 4:**
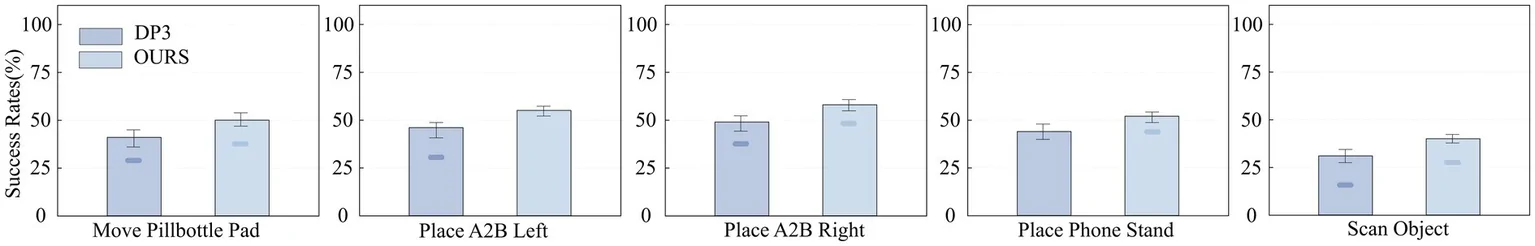
Comparative analysis of success rate performance across the Aloha-AgileX platform.

Table 5 provides an additional evaluation of the proposed approach in comparison with multiple classical methods. All models were assessed under uniform experimental conditions, including identical datasets, hardware platforms, and inference environments. For each configuration, several test rounds were conducted, each comprising 100 independent inference runs. The final success rates were computed by averaging the results across all rounds to guarantee statistical robustness.

**Table 5:** Success rate (%) comparison across different methods and tasks.

Method	Move pill bottle pad	Place A2B left	Place A2B right	Place Phone stand	Scan object
Pi0	21% ± 4	31% ± 5	27% ± 5	35% ± 5	18% ± 4
ACT	0% ± 0	1% ± 1	0% ± 0	2% ± 1	2% ± 1
DP	1% ± 0	2% ± 1	13% ± 3	13% ± 3	9% ± 3
DP3	41% ± 4	46% ± 5	49% ± 4	44% ± 5	31% ± 4
OURS	50% ± 3	55% ± 4	58% ± 3	52% ± 4	39% ± 3

**Statistical reporting:** All results reported in Table 5 and Figure 4 are averaged over 5 independent training seeds, with 100 evaluation rollouts performed per trained model. Error bars in Figure 4 represent standard deviation across the 5 training seeds. For each seed, 100 episodes are evaluated with randomly initialized object configurations and report the mean success rate; the final value is the average across all seeds. To assess the statistical significance of improvements over the strongest baseline DP3, paired two-tailed *t*-tests are performed across the 5 seeds for each task. The improvements are statistically significant with p<0.05 for all tasks, and p<0.01 for Place A2B Left, Place A2B Right, and Place Phone Stand, confirming the robustness of the method.

**Baseline implementation and performance:** All baselines were implemented using official codebases with recommended hyperparameters and trained for the same number of updates on identical data. ACT and Diffusion Policy (DP) perform poorly in the bimanual setting due to: (1) reliance on 2D RGB observations lacking 3D geometric information critical for dual-arm coordination, (2) reactive architectures ill-suited for long-horizon tasks, and (3) the high-dimensional action space (14 DoF). Notably, WIS and DP3 utilize 3D point clouds, while ACT and DP use RGB images only; thus, the comparison reflects representation-policy combinations rather than purely architectural differences. The “Pi0” baseline refers to the $\pi_{0}$ model from the original RoboTwin2.0 benchmark, using the official pre-trained checkpoint with multi-view RGB and proprioceptive inputs.

**Ablation studies:** Comprehensive ablation experiments are conducted on the Place A2B Right task to systematically investigate each architectural component in WIS. Six ablation variants isolate the key design choices: All ablation variants use identical hyperparameters as the full WIS model. Each variant is evaluated over 100 rollouts with multiple random seeds.

Table 6 presents the ablation results with several key observations. The results indicate that every architectural element plays a non-trivial role, with the world model and cross-modal attention mechanisms exhibiting particularly pronounced effects. The guidance gradient and high-level planner also provide clear performance gains, although they are comparatively smaller. Additionally, a variant relying solely on world conditioning underperforms the full model, underscoring the importance of integrating multiple complementary design choices.

**Table 6:** Ablation study results on the place A2B right task.

Variant	Success rate (%)	Δ vs. full WIS
Full WIS	58% ± 3	-
w/o world model	44% ± 3	−14
w/o guidance gradient	49% ± 2	−9
w/o high-level planner	50% ± 2	−8
w/o cross-modal attention	46% ± 3	−12
World conditioning only	47% ± 2	−11
Single-level controller	45% ± 3	−13

## 5 Conclusion

This study proposes WIS, a hierarchical framework for bimanual robotic manipulation that integrates a language-driven world model with a diffusion-based policy to enable predictive reasoning in action generation. The approach in this study combines three key modules: a cross-modal perception module for semantic grounding, a language-conditioned world model for future state forecasting, and a hierarchical diffusion policy with prediction-aware sampling that actively incorporates anticipated outcomes into action refinement. Extensive experiments on the RoboTwin 2.0 platform using Aloha-AgileX robots demonstrate that WIS consistently outperforms strong baseline methods across multiple long-horizon bimanual tasks, achieving superior success rates with only marginal increases in computational overhead. These results validate that explicit predictive reasoning, tightly coupled with semantic understanding and hierarchical generation, offers an efficient and robust solution for temporally coherent dual-arm manipulation.

### 5.1 Limitations

The prediction-aware guidance relies on a reference future distribution derived from demonstrations, which may not encompass all viable strategies. Currently, the evaluation is simulation-only; physical deployment would introduce perception noise, actuation delays, and calibration errors. The high-level latent plan lacks explicit subgoal supervision, and its semantic interpretability remains unexplored. Although the sensitivity analysis confirms the plan’s influence, further visualization and semantic analysis of the latent space are deferred to future research. Moreover, the evaluation only tests in-distribution generalization across varied initial configurations and object appearances; the study does not assess generalization to new task semantics, unseen object categories, altered viewpoints, or paraphrased instructions. Future work should explore uncertainty-aware planning, real-system transfer, and online adaptation.

## Data Availability

The datasets presented in this article are not readily available because the data used in this study were generated using the RoboTwin 2.0 simulation platform through its automated task execution pipeline. The datasets consist of expert demonstration trajectories synthesized under domain-randomized settings and were generated specifically for the experiments reported in this paper. The data are available from the corresponding author upon reasonable request for research and verification purposes. Requests to access the datasets should be directed to lxk14012@foxmail.com.
